# Buffering and Amplifying Interactions among OAW (Ocean Acidification & Warming) and Nutrient Enrichment on Early Life-Stage *Fucus vesiculosus* L. (Phaeophyceae) and Their Carry Over Effects to Hypoxia Impact

**DOI:** 10.1371/journal.pone.0152948

**Published:** 2016-04-04

**Authors:** Balsam Al-Janabi, Inken Kruse, Angelika Graiff, Vera Winde, Mark Lenz, Martin Wahl

**Affiliations:** 1 GEOMAR, Helmholtz Centre for Ocean Research Kiel, Benthic Ecology Group, Kiel, Germany; 2 University of Rostock, Institute of Biological Sciences, Applied Ecology and Phycology, Rostock, Germany; 3 Leibniz Institute of Baltic Sea Research, Geochemistry & Isotope Biogeochemistry Group, Department of Marine Geology, Warnemünde, Germany; University of California Santa Cruz, UNITED STATES

## Abstract

Ocean acidification and warming (OAW) are occurring globally. Additionally, at a more local scale the spreading of hypoxic conditions is promoted by eutrophication and warming. In the semi-enclosed brackish Baltic Sea, occasional upwelling in late summer and autumn may expose even shallow-water communities including the macroalga *Fucus vesiculosus* to particularly acidified, nutrient-rich and oxygen-poor water bodies. During summer 2014 (July–September) sibling groups of early life-stage *F*. *vesiculosus* were exposed to OAW in the presence and absence of enhanced nutrient levels and, subsequently to a single upwelling event in a near-natural scenario which included all environmental fluctuations in the Kiel Fjord, southwestern Baltic Sea, Germany (54°27 ´N, 10°11 ´W). We strove to elucidate the single and combined impacts of these potential stressors, and how stress sensitivity varies among genetically different sibling groups. Enhanced by a circumstantial natural heat wave, warming and acidification increased mortalities and reduced growth in *F*. *vesiculosus* germlings. This impact, however, was mitigated by enhanced nutrient conditions. Survival under OAW conditions strongly varied among sibling groups hinting at a substantial adaptive potential of the natural *Fucus* populations in the Western Baltic. A three-day experimental upwelling caused severe mortality of *Fucus* germlings, which was substantially more severe in those sibling groups which previously had been exposed to OAW. Our results show that global (OAW), regional (nutrient enrichment) and local pressures (upwelling), both alone and co-occurring may have synergistic and antagonistic effects on survival and/or growth of *Fucus* germlings. This result emphasizes the need to consider combined stress effects.

## Introduction

Global climate change will expose marine populations to increased eutrophication and upwelling events at a regional scale and to ocean acidification and warming (OAW) at a more global scale [1]. For the Baltic Sea, current models predict an increase in pCO_2_ from currently almost 400 μatm to 1000 μatm, an increase in sea surface temperature by up to 5°C and considerable enhancements of eutrophication and hypoxia during the next 100 years [2]. The combined effects of these global change factors will likely affect Baltic ecosystems [3]. Anthropogenic activities, such as agriculture, lead to eutrophic conditions in the largest part of the Baltic Sea, including the southwestern part with Kiel Bight [4]. Eutrophication may further intensify due to increased precipitation and river runoff [5, 6]. Hypoxic conditions (< 2 mg O_2_ L^-1^) are predicted to increase in the Baltic Sea during this century as modelled by Meier and Andersson [7]. In Kiel Bay, seasonal oxygen depletion has been known for decades [8], but is likely to intensify further.

Macrophytes play a key role in ecosystem services by the retention of excessive nutrients [9, 10], acting as ‘nutrient filters’ [11] and providing the benthic ecosystem with oxygen [12]. A combination of global, regional and local stressors endangers macroalgae worldwide [13, 14]. Among these, the bladder wrack *Fucus vesiculosus* is a dominant perennial macroalga in the intertidal and shallow subtidal of the Western, Central and Eastern Baltic Sea [15]. As a foundation species, *F*. *vesiculosus* provides habitat and food for a large variety of invertebrate assemblages [16, 17]. A drastic decline in *F*. *vesiculosus* during the last five decades was observed, e.g. by almost 95% in Kiel Bay, Western Baltic Sea [18, 19]. The shoaling of *F*. *vesiculosus* was mainly assigned to the severe indirect effects of eutrophication [18, 20–22]. These are *i*.*a*. the increased turbidity [20], competition with ephemeral algae [23] and increased palatability [24]. Also, enhanced sedimentation reduces the attachment and survival of *F*. *vesiculosus* zygotes [25, 26]. Regarding direct effects, elevated nutrient concentrations positively affect photosynthesis and growth in adult *F*. *vesiculosus* [27].

As a consequence of eutrophication in the Baltic Sea, sedimentation and bacterial re-mineralization are enhanced, leading to seasonal hypoxia and hypercapnia in sub-surface waters [28]. Further spread of (periodic) hypoxic areas in the Baltic Sea is predicted until the end of the 21^st^ century due to the interplay between eutrophication and lower oxygen solubility under warming [7]. Hypoxia induced mortality of benthic organisms was reviewed by Gray et al. [29] and recognized at a Baltic [30] and a global scale [31]. Also, higher frequencies of local upwelling in the last decades have been documented in the Baltic Sea [32].

In addition to hypoxia, warming was observed to impact growth, survival and photosynthetic efficiency in *F*. *vesiculosus*, while acidification showed weaker effects in the early [33] and adult life-stage [34]. As a consequence of warming, poleward range shifts of seaweed populations have been observed worldwide [14, 35, 36]. Also in the Baltic Sea, northward range shifts of *F*. *vesiculosus* populations have been observed and are predicted to continue during the 21^st^ and 22^nd^ century [37]. In contrast, ocean acidification was reported to increase growth in non-calcifying macroalgae [27, 38]. Assumed reasons are enhanced availability of CO_2_ to saturate the carbon demand during photosynthesis or the saved energy when carbon concentrating mechanisms are downregulated [39]. However, physiological responses of the macroalga *Macrocystis pyrifera* to acidified conditions showed that increased CO_2_ conditions did not affect growth or photosynthesis [40]. Early life-stage macroalgae may be particularly threatened by global change [41]: Warming lowers the germination success of *F*. *vesiculosus* at 25°C [42] and reduces survival in *F*. *serratus* germlings more severely than in adults [43]. Enhanced sedimentation caused by eutrophication is more detrimental for young [23, 44] than for adult *Fucus* [15, 45]. Despite recognized differences in stress sensitivity among life-stages of a species, past research has mainly focussed on adult forms [46, 47].

Adaptation of marine populations to global change is favoured by genetic diversity [48, 49]. Conversely, low intraspecific genetic diversity and high phenotypic plasticity and gene flow restrict adaptation to environmental stress [50–53]. The increased tolerance of the eelgrass *Zostera marina* with higher genetic diversity to a summer heat wave [54] showed that genetic diversity may buffer warming stress at the population level [55].

Baltic *F*. *vesiculosus* populations show reduced genetic variation compared to Atlantic populations probably due to isolation and bottlenecks as well as the eroded genetic variation due to selection [56]. It has been argued that environmental stress, e.g. osmotic stress in the brackish Baltic, lower genetic diversity and limited dispersal capacity of *F*. *vesiculosus* gametes [57] may favour local extinctions [58]. However, this study is the first one to test genetic variation in Baltic *F*. *vesiculosus* with regard to the sensitivity towards OAW, nutrient enrichment and subsequent hypoxia.

The aim of the present study was to investigate (1) how OAW, (2) nutrient enrichment and (3) upwelling events affect the survival and growth of *F*. *vesiculosus* germlings, (4) how OAW interacts with simultaneous exposure to high nutrient concentrations, (5) how these treatments modify hypoxia sensitivity and (6) whether sibling groups vary in their tolerance towards these environmental parameters. Our experimental concept, hence, consisted in the exposure of genetically different sibling groups of *F*. *vesiculosus* germlings to increased temperature, pCO_2_ and nutrient conditions and to a final upwelling event while maintaining the natural variations.

## Material and Methods

### Collection, gamete acquisition, experimental design

A total of 64 fertile *F*. *vesiculosus* were collected in a wave exposed area with mixed hard substrate and sand bottom in the southwestern Baltic Sea (Bülk, Germany, 54°27.327 ´N, 10°11.977 ´W) in mid-June 2014. To avoid the collection of siblings and ensuring for genetic variability, individuals sampled were distanced by at least 2 meters, which is the estimated maximum dispersal distance of most *F*. *vesiculosus* gametes [57]. After collection, algae were transported to the lab in cooler boxes. Fertile receptacles were cut from these dioecious algae and gender was determined (46 females, 18 males) under the microscope at 100 x magnification (Olympus BH-2). Receptacles were rinsed with tap water, blotted dry and stored in the dark for 6 days at 14°C. Before gamete release, all receptacles from one female and one male individual (i.e. one parental pair) were put in a small dish. Gamete release followed by egg fertilisation was induced by immerging receptacles into sand-filtered seawater (15–16 psu) and exposing them to light (110 μmol photons m^-2^ s^-1^) for 3 hours. In this way, gametes were obtained from16 parental pairs. No specific permits were required for this study, the location is not privately-owned or protected and the study did not involve an endangered or protected species.

One mL of homogeneously suspended fertilised eggs was pipetted onto the upper surface of each of 2 x 2 cm sandstone cubes. Each cube with its settled germlings represented one experimental population. 16 different populations, each composed of germlings stemming from one parental pair, were thus produced. Culture and monitoring of germlings took place in a room with windows approximating natural light conditions during 3 weeks with weekly water exchange (15–16 psu) at 15°C until introducing them to the Kiel Outdoor Benthocosms (KOB). This facility maintains the natural *in situ* fluctuations and simultaneously allows manipulating environmental variables (e.g. temperature, pCO_2_) on top of these fluctuations, i.e. “delta treatments”. Target temperatures were obtained and maintained by computer controlled heaters and coolers, while acidification was achieved by increasing the atmospheric pCO_2_ within the enclosed head space above the tanks by injecting pre-mixed gas to maintain an atmospheric pCO_2_ of 1100 μatm. Details of the experimental set-up of the KOB and the logged conditions in the tanks are given in Wahl et al. [59].

### OAW x nutrient and upwelling treatment

The OAW x nutrient experiment took place during 2 summer months from mid-July until mid-September 2014. One PVC box (70 cm x 40 cm x 12 cm) was placed within each of the 12 Benthocosm main tanks, each of them containing all 16 experimental populations of germlings. Since the upper rim of the lid-less boxes was a few centimetres above the water surface, the water body within the PVC boxes was separated from the water body of the main tank but open to the atmosphere. Thus, the boxes experienced the same treatments as the main tank regarding OAW but were insulated from gene flow from the adult *Fucus* population in the main tank. Twice a week the water of the PVC box was exchanged by water from the main tank which had been filtered through a 50 μm mesh to prevent the introduction of *F*. *vesiculosus* eggs (100 μm diameter). As the single-factor and combined effects of temperature and pCO_2_ on *F*. *vesiculosus* germlings have been investigated previously [33], we combined warming and acidification into a single factor (OAW) in the present run. The two fully crossed factors OAW and nutrients were applied at two levels each (ambient and future). Ambient and predicted future levels of OAW and nutrients were simulated by adding the expected shift to the natural fluctuations of the ambient fjord conditions as delta treatments [59].

The “ambient” condition represents the natural fjord conditions transported into the main tanks of the KOB by a continuous flow-through (1 tank-volume per day, i.e. 1500 L/ 24 h) of Kiel Fjord water pumped from 1 m depth. “Future” conditions were simulated by adding 5°C to the actual temperature of the Kiel Fjord and by increasing the pCO_2_ concentration in the hooded headspace of the tanks to 1100 μatm according to the predictions for the year 2110 for the Baltic Sea [2]. The bi-weekly nutrient enrichment (2x ambient) was achieved by doubling the “ambient” concentration which was taken as the seven years (2006–2013) mean for each specific date of nutrient addition (Table 1, S1 Fig). NaNO_2_ (Merck, Germany), NaNO_3_ (Carl Roth, Germany) and H_2_NaO_4_P.H_2_O (ACROS organics, Germany) were dissolved in fjord water 10 minutes before addition to the nutrient treatments. The ratio P: N of the Kiel Fjord is approximately 1: 1.5 and does not match the Redfield Ratio, probably due to the nutrient input of the nearby located river Schwentine. Additionally, high organism activity during summer months decreases the overall nutrient availability in shallow water. This ambient P: N ratio was not altered when NO_3_, NO_2_ and PO_4_ concentrations (μmol L^-1^) were doubled under “future” conditions. Our analysis on CN ratio in *Fucus* tissue (see result section below) showed no differences under “future” conditions, indicating that carbon did not become a limiting factor under nutrient enrichment.

**Table 1:** Nutrient concentration under ambient and future conditions. “Ambient” nutrient concentrations of PO_4_, NO_2_, NO_3_ (μmol L^-1^) for the respective summer months and “future” nutrient concentrations (μmol L^-1^).

	July		August		September	
	Ambient	Future	Ambient	Future	Ambient	Future
PO_4_	0.46	0.93	0.59	1.19	1.06	2.11
NO_3_	0.53	1.05	0.77	1.54	1.27	2.54
NO_2_	0.18	0.36	0.20	0.40	0.22	0.44

The four treatment combinations, each replicated three times, thus were: OAW- N- (ambient OAW & ambient nutrients), OAW- N+ (ambient OAW & high nutrients), OAW+ N- (future OAW & ambient nutrients) and OAW+ N+ (future OAW & high nutrients). These treatment combinations were regularly distributed among the 12 experimental units.

The upwelling experiment was performed immediately after the end of the OAW x nutrient treatment phase, i.e. when all the treatments in the tanks were set back to "ambient". During three days, hypoxic fjord water from 15 m depth (O_2_ = 2.75 ± 0.41 mg L^-1^, T = 16.52 ± 0.33°C, pH = 7.40, Sal = 22.8) was pumped as flow-through into the KOB and, via a bypass into the germlings boxes continuously during 3 days. This experiment was meant to assess the effect of the compound factor "upwelling" on sibling groups pre-conditioned by the foregoing treatment combinations (OAW x nutrient enrichment). Upwelled water in this region and this season usually is characterized by lower oxygen, higher salinity and higher nutrients than surface water.

### Measurement of abiotic factors

pH and temperature of the main tank of the KOB were measured daily with a calibrated hand-held sensor (pH, Mettler Toledo GmbH, Giessen, Germany) while oxygen was measured with a Multi WTW Oxy 3515 (oxygen, Wissenschaftlich Technische Werkstätten, Weilheim, Germany). Salinity was measured with a portable conductivity meter (WTW Cond 3110 + Tetra Con 325, Wissenschaftlich Technische Werkstätten, Weilheim, Germany). Additionally, temperature, pH, O_2_ and salinity were continuously logged at 10 min time intervals (GHL Advanced Technology, Kaiserslautern, Germany). During the hypoxia experiment, O_2_ and temperature within the PVC boxes were logged every 10 min using the Multi WTW Oxy 3515. Samples for nutrient concentrations were taken from the KOB before each water exchange in the PVC boxes as initial concentration. For this, water samples were immediately filtrated through a 0.45 μm Minisart syringe filter (Sartorius) in 10 mL tubes, stored at -20°C and measured with a QuAAtro nutrient analyzer [60] (SEAL Analytical; S1 Fig). More details about the measurement of the abiotic variables in the KOB are described by Wahl et al. [59].

### Abiotic conditions

During the OAW x nutrient experiment, the bi-weekly measured oxygen concentrations (mean = 8.91 ± 0.38 mg L^-1^) varied in the low OAW treatment with a minimum of 8.01 ± 0.23 mg L^-1^ and a maximum of 9.44 ± 0.16 mg L^-1^ (mean ± SD) and within the high OAW treatment (mean = 7.76 ± 0.56 mg L^-1^) with a minimum of 6.75 ± 0.56 mg L^-1^ and a maximum of 8.72 ± 0.21 mg L^-1^. Day-night fluctuations measured in two hours intervals showed that O_2_ concentrations varied between a minimum of 7.64 mg L^-1^ at 4:30 am and a maximum of 9.97 mg L^-1^ at 16:30 pm under ambient conditions.

**Fig 1:**
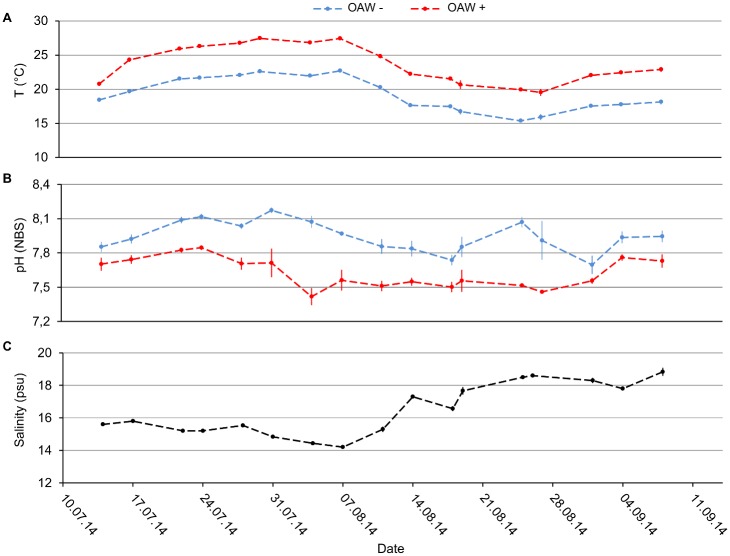
Temperature, pH and salinity during the OAW x nutrient experiment. (**A**) Temperature (°C), (**B**) pH (NBS, National Bureau of Standards) under the two treatment combinations ambient (OAW-), warmed and acidified (OAW+) and (**C**) salinity (psu) at the ambient treatment (OAW-). Data are the mean ±SD of three replicates in each treatment combination.

Ambient temperatures (factor level ‘ambient’) (mean = 19.26 ± 2.38°C) varied between bi-weekly measurements with a minimum of 15.37 ± 0.19°C and a maximum of 22.7 ± 0.08°C. Elevated temperatures (factor level ‘future’) (mean = 23.64 ± 2.68°C) varied between a minimum of 19.53 ± 0.52°C and a maximum of 27.47 ± 0.26°C, which occurred during a natural summer heat wave (mean ± SD) (Fig 1). Temperatures during day and night fluctuated between a minimum of 16.1°C at 10:30 pm and a maximum of 17.7°C at 8:30 am under ambient conditions.

### Response variables

#### Growth

For growth measurements, digital images were taken of 10–15 randomly chosen individual germlings per population at 40 x magnification (SteREO Discovery. V8 –Carl Zeiss Jena GmbH) similar to Steen and Scrosati [61]. Measured individuals were chosen randomly, since germlings were too small for labelling. The projected side-view of the single germlings was measured with the image analysis software Image J 1.45s (National Institutes of Health, USA) and the mean of germlings’ area of the perpendicular projection was calculated for each population. Germlings’ area was measured at the beginning (area t_0_) and after 8 weeks (area t) at the end of the OAW x nutrient experiment. Relative growth rate (RGR) in % d^-1^ was calculated as exponential growth:

$$RGR=\;\left[{\left(\frac{Area\;t}{Area\;t_{0}}\right)}^{1/\Delta t}-1\right]\;.\;100$$

Where *Δt* is the time period between t_0_ and t in days.

#### Survival

Germling number was counted under a binocular at 25x magnification between the start (number t_0_) and after 8 weeks (number t) of the OAW x nutrient experiment. Survival of germlings was expressed as the percent of surviving germlings and calculated as:

$$Survival\;\text{\%}=\frac{Number\;t}{\;Number\;t_{0}}\;.\;100$$

For determining survival during the final upwelling experiment, the germling number at the end of the preceding OAW x nutrient experiment was set as *t*_*0*_ and number *t* was the germling number after the upwelling treatment.

#### Log-effect ratio

Log effect ratios were performed to show the direction and the strength of the sibling groups’ phenotypical responses to the different OAW and nutrients enhancements relative to the respective ambient conditions. These differences among sibling groups’ responses were determined in order to assess whether higher genetic diversity also increases the variance in responses. Sibling groups’ sensitivity to high OAW and N were calculated separately as the log effect ratios of growth or survival under future relative to actual conditions, as:

$$log\;effect\;ratio\;OAW=\text{log}\left(\frac{Growth\;OAW+}{Growth\;OAW-}\right)$$

at ambient and high nutrient conditions and:

$$log\;effect\;ratio\;N=\text{log}\left(\frac{Growth\;N+}{Growth\;N-}\right)$$

at ambient and high OAW. The same procedure was used for calculating the log effect ratios for survival. Negative growth rates observed in 3 populations were attributable to the mortality of the bigger sized individuals and a decrease in mean area t compared to t_0_. To avoid negative values for log effect ratio analysis, growth was measured as ratios of area t and area t_0_ as:

$$Growth\;=\;\frac{Area\;t}{Area\;t_{0}}$$

#### CN ratios

Germlings of five sibling groups (1, 3, 7, 12 and 14) of the two treatment combinations OAW- N- and OAW- N+ were pooled for CN analysis. For the analysis of carbon and nitrogen content, freeze-dried algal material was ground to powder and three subsamples of 2 mg from each treatment was packed and loaded into tin cartridges (6×6×12 mm). Then, the packages were combusted at 950°C and the absolute C and N contents in % dry weight (% DW) were automatically quantified in an elemental analyser (Elementar Vario EL III, Germany) using acetanilide as standard according to Verardo et al. [62].

### Statistical Analysis

Growth (RGR, % d^-1^) and survival (%) were analysed using a split-plot ANOVA with the fixed factors ‘OAW’ (with two levels: OAW- and OAW+) and ‘Nutrients’ (with two levels: N- and N+) as well as the random factors ‘Mesocosm’ (with 12 levels) and ‘Sibling Group’ (with 16 levels). ‘Mesocosm’ was nested in the ‘OAW’ x ‘Nutrients’ interaction. This model allowed us to analyse the influence of the fixed factors. Furthermore, we were able to identify possible random-by-fixed factor interactions between ‘Sibling Group’, ‘OAW’ and ‘Nutrients’. Survival data were arcsine transformed prior to the analysis to overcome data truncation. Differences in carbon and nitrogen contents (% DW) as well as in CN ratios between five single sibling groups were identified in a further split-plot ANOVA with the fixed factor ‘Nutrients’ (two levels: see above), which was combined with OAW only, and the random factors ‘Mesocosm’ (12 levels) and ‘Sibling Group’ (16 levels), while ‘Mesocosm’ was nested in ‘Nutrients’. Split-plot ANOVAs were performed by using Satterthwaite’s method for denominator synthesis [63], which calculates appropriate error terms for the F-ratios of the respective effects. Normality of errors and homogeneity of variances were verified by using residual plots (Q-Q Plot and Standardized Residuals Plot, respectively) in STATISTICA and R. The significance level of all analyses was α = 0.05. Post-hoc tests were performed using Tukey’s HSD. All statistical analyses were conducted using the software STATISTICA v. 12 [64] and R.

## Results

### OAW x nutrient experiment (mid-July–mid-September 2014)

#### Growth and survival

**Fig 2:**
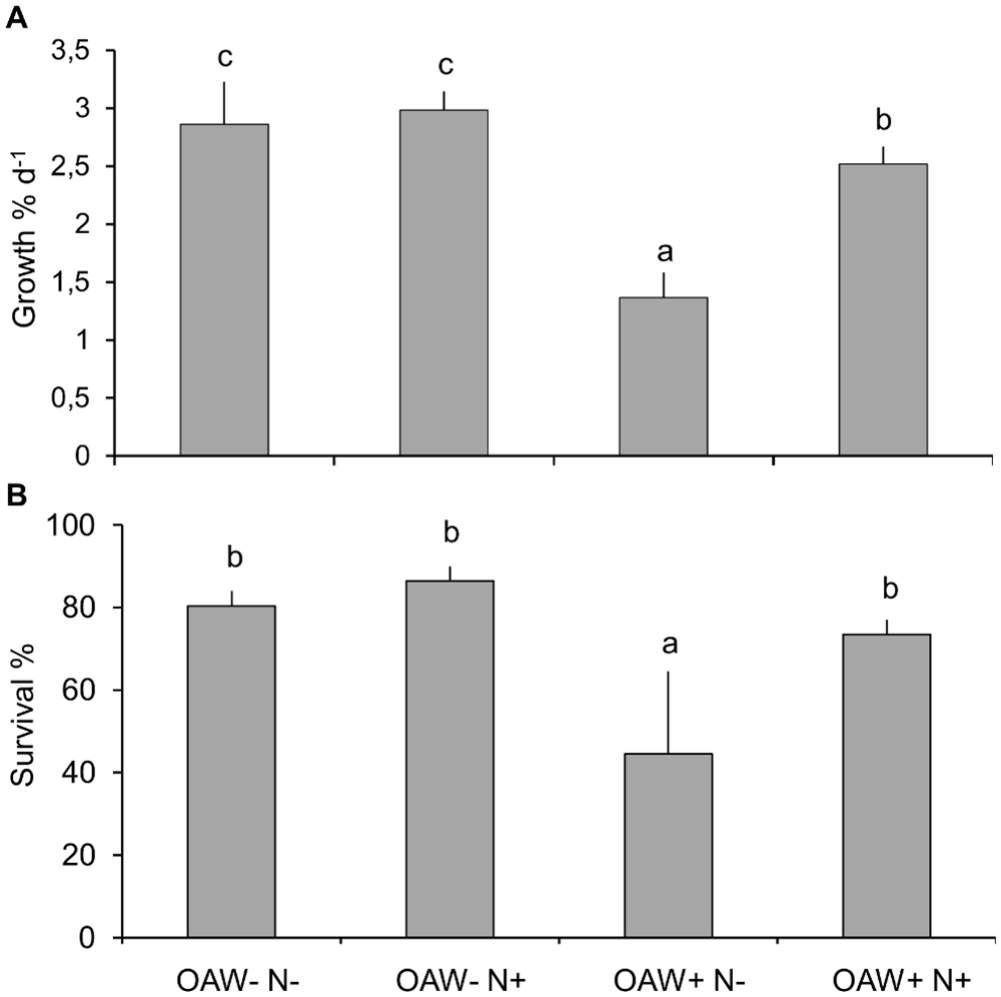
Growth and survival of *F*. *vesiculosus* germlings during the OAW x nutrient experiment. (A) Growth (% d^-1^) and (B) survival (%) (mean +SD, n = 3, 8–9 weeks) during summer 2014 at the four treatment levels OAW-N-, OAW-N+, OAW+N-, OAW+N+. Means were calculated from 16 sibling groups for each treatment ‘OAW’ and ‘Nutrient’ treatment combination. Different letters above the bars indicate significant differences (p-value < 0.05) between the treatments after Tukey’s HSD.

Under ambient temperature, pCO_2_ and nutrient conditions, *F*. *vesiculosus* germlings’ relative growth rate (RGR) and survival was 2.86 ± 0.37% d^-1^ (Fig 2A) and 80.34 ± 3.70% (Fig 2B), respectively. Under ambient temperature and pCO_2_ conditions, the addition of nutrients (i.e. OAW-N+) did not change RGR and survival (Fig 2). In contrast, high OAW conditions under ambient nutrient conditions (OAW+N-) reduced RGR and survival by about 50%; when RGR was 1.37 ± 0.21% d^-1^ and survival was 44.48 ± 20.03% (mean, ± SD, n = 3, Table 2). The addition of nutrients almost entirely compensated the negative impact of OAW as reflected in a RGR of 2.52 ± 0.15% d^-1^ and survival of 73.49 ± 3.49%. The significant interaction between the factors OAW and nutrients regarding growth rate reflects this compensation effect of the nutrient treatment (Split-plot ANOVA, p < 0.05, Table 2A).

**Table 2:** OAW and nutrients effects on growth and survival. Results from split-plot ANOVA with the fixed factors ‘OAW’ and ‘Nutrients’ and the random factors ‘Mesocosm’ and ‘Sibling group’. Effects are shown for (A) growth rates (% d^-1^) and (B) survival during the OAW x nutrient experiment and (C) survival during the upwelling experiment. Df: degrees of freedom, SS: sums of squares and MS: mean squares. ‘Den. Syn. Error df’ and ‘Den. Syn. Error MS’ describe the denominator synthesis of degrees of freedom and mean squares, respectively. (Datasets of area and growth values can be found in the PANGAEA dataset).

Source of variation	*df*	*SS*	*MS*	*Den*. *Syn*. *Error df*	*Den*. *Syn*. *Error MS*	*F-value*	*p-value*
(A) Growth rates OAW x nutrient experiment							
OAW	1	47.659	47.659	6.156	1.188	40.111	< 0.001
Nutrients	1	20.468	20.468	8.469	1.428	14.335	< 0.05
OAW x Nutrients	1	13.473	13.473	8	1.339	10.055	0.013
Mesocosm (OAWxNutrients)	8	10.720	1.340	135	0.389	3.443	0.001
Sibling	15	27.120	1.808	5.276	0.325	5.558	0.030
Sibling x OAW	15	3.561	0.237	135	0.389	0.610	0.863
Sibling x Nutrient	15	7.156	0.477	135	0.389	1.226	0.260
Error	135	52.542	0.389				
(B) Survival OAW x nutrient experiment							
OAW	1	4.069	4.069	14.366	0.867	4.694	0.048
Nutrients	1	2.903	2.903	7.061	0.513	5.656	0.049
OAW x Nutrients	1	1.272	1.272	8	0.535	2.376	0.162
Mesocosm (OAWxNutrients)	8	4.281	0.535	135	0.163	3.284	0.002
Sibling	15	11.786	0.786	12.535	0.473	1.662	0.186
Sibling x OAW	15	7.420	0.495	135	0.163	3.036	< 0.001
Sibling x Nutrient	15	2.117	0.141	135	0.163	0.866	0.603
Error	135	21.998	0.163				
(C) Survival Upwelling experiment							
OAW	1	4.907	4.907	8.398	0.550	8.914	0.017
Nutrients	1	0.322	0.322	5.284	0.423	0.761	0.421
OAW x Nutrients	1	2.913	2.913	8	0.516	5.647	0.045
Mesocosm (OAWxNutrients)	8	4.127	0.516	135	0.164	3.150	0.003
Sibling	15	10.711	0.714	3.504	0.105	6.793	0.052
Sibling x OAW	15	2.976	0.198	135	0.164	1.212	0.270
Sibling x Nutrient	15	1.057	0.070	135	0.164	0.430	0.968
Error	135	22.106	0.164				

#### Sibling groups differences in growth

**Fig 3:**
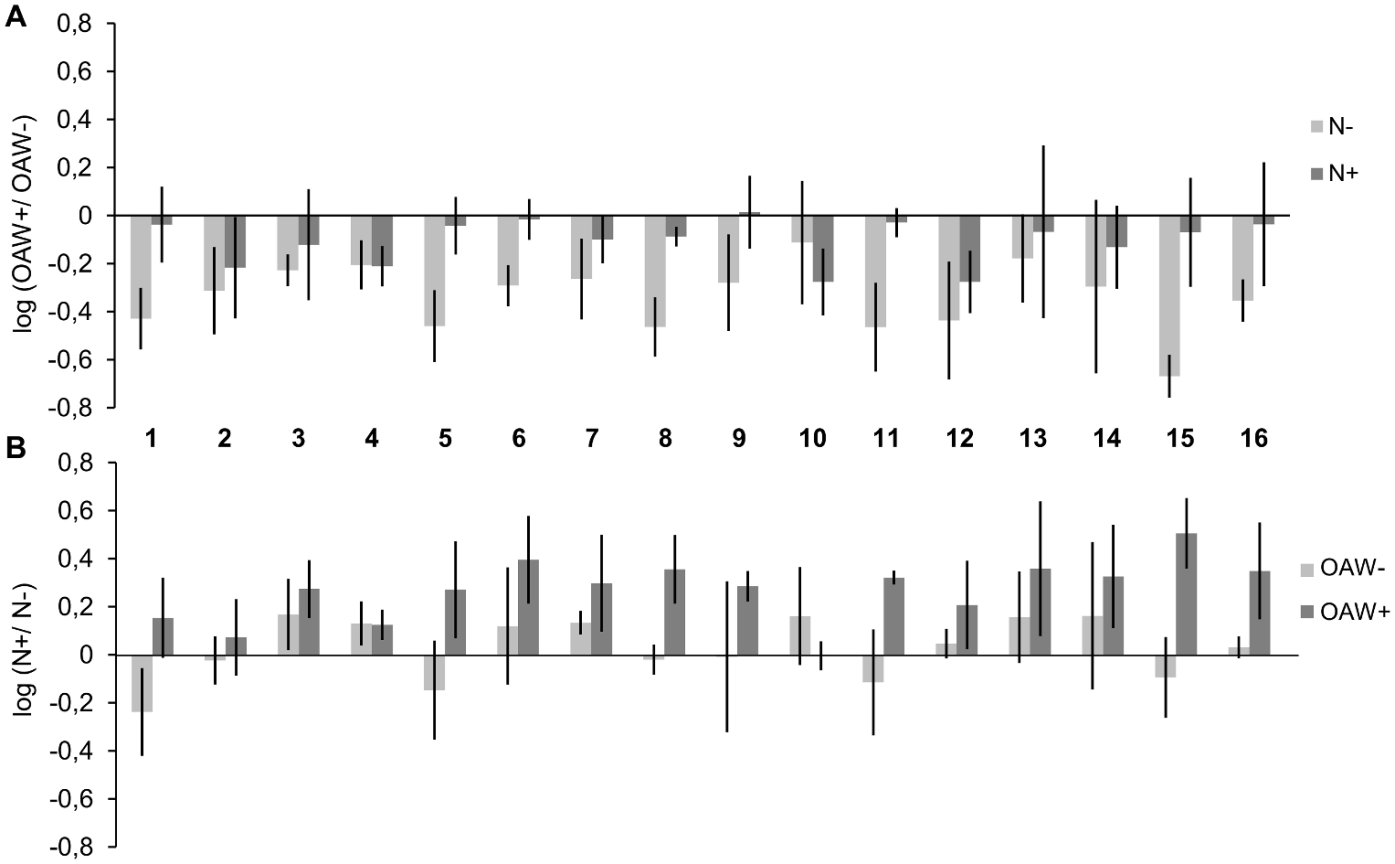
Log effect ratios of growth in 16 sibling groups during the OAW x nutrient experiment. Log effect ratios (mean ± 95% confidence intervals, n = 3) of (A) OAW effects were calculated as log (growth OAW+/ growth OAW-) under ambient and high nutrient conditions (N-, N+) and (B) log effect ratios for nutrient effects calculated as log (growth N+/ growth N-) at ambient and high OAW (OAW-, OAW+).

RGR of the 16 sibling groups differed significantly (Split-plot ANOVA, factor: sibling group, p-value < 0.05, Table 2A). Nutrient enrichment at ambient temperature and CO_2_ enhanced growth significantly only of the sibling groups 3, 4 and 7 (Fig 3B). In contrast, nutrient enrichment under high OAW conditions enhanced growth in most sibling groups, notably in the groups 3, 4, 5, 6, 7, 8, 9, 11, 12, 13, 14, 15 and 16. High OAW at ambient nutrient conditions decreased growth in most sibling groups, i.e. 1, 2, 3, 4, 5, 6, 7, 8, 9, 11, 12, 15 and 16. In contrast, under nutrient enrichment the negative effect of high OAW was mitigated so that growth was significantly decreased only in the 6 sibling groups 2, 4, 7, 8, 10 and 12 (Fig 3A). The factor ‘sibling group’ did not interact significantly with ‘OAW’ or with ‘nutrients’ (Split-plot ANOVA, sibling group x OAW, sibling group x nutrients, p-value > 0.05, Table 2A).

#### Sibling groups differences in survival

**Fig 4:**
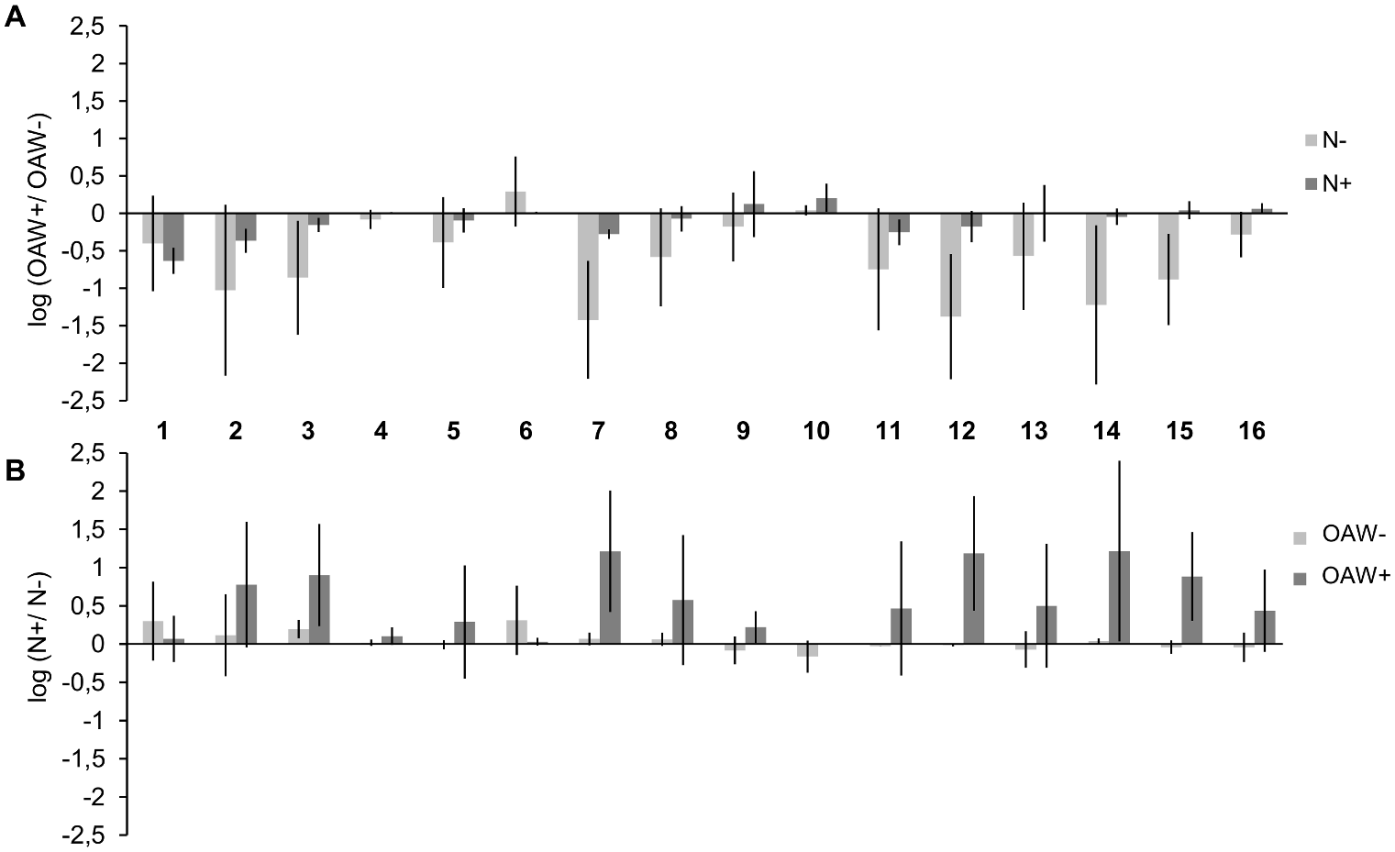
Log effect ratios of survival in 16 sibling groups during the OAW x nutrient experiment. Log effect ratios (mean ± 95% confidence intervals, n = 3) of (A) OAW effects were calculated as log (survival OAW+/ survival OAW-) under ambient and high nutrient conditions (N-, N+); (B) log effect ratios for nutrient effects were calculated as log (survival N+/ survival N-) at ambient and high OAW (OAW-, OAW+).

OAW+ at ambient nutrient concentrations decreased survival significantly in 5 out of 16 sibling groups, notably 3, 7, 12, 14 and 15. The different survival responses to increased OAW of sibling groups are reflected in the significant interaction of the factors ‘OAW’ x ‘Sibling group’ (Split-plot ANOVA, p-value < 0.001, Table 2B). Nutrient enrichment tended to mitigate the negative effect of OAW and this buffering influence was significant in the 4 sibling groups 7, 12, 14 and 15 (Fig 4A). Nutrient enrichment under ambient temperatures and CO_2_ did not affect sibling groups’ survival. In contrast, nutrient enrichment under warming and acidification generally tended to improve survival and significantly enhance survival in the groups 3, 7, 12 and 15 (Fig 4B, Table 2B).

#### CN ratio

Germling carbon and nitrogen content (% DW) as well as the CN ratio did not differ significantly among the nutrient treatments (Split-plot ANOVA, p-value > 0.05, Table 3). The CN ratio was 10.56 ± 1.13 under ambient nutrient conditions and 7.18 ± 3.06 (mean ± SD) under high nutrient conditions.

**Table 3:** Nutrient effect on carbon and nitrogen content (% DW) and on the CN ratio. Split-plot ANOVA with the fixed factor ‘Nutrient’ and the random factors ‘Mesocosm’ and ‘Sibling group’. Effects of the nutrient treatment were analysed for (A) the carbon content (% DW), (B) the nitrogen content (% DW) and (C) the CN ratio. Df: degrees of freedom, SS: sums of squares and MS: mean squares. ‘Den. Syn. Error df’ and ‘Den. Syn. Error MS’ describe the denominator synthesis of degrees of freedom and mean squares, respectively.

Source of variation	*df*	*SS*	*MS*	*Den*. *Syn*. *Error df*	*Den*. *Syn*. *Error MS*	*F-value*	*p-value*
(A) Carbon content							
Nutrients	1	36.563	36.563	1.998	17.839	2.05	0.289
Mesocosm (Nutrients)	4	79.878	19.969	16	15.482	1.290	0.315
Sibling	4	145.584	36.396	4	13.352	2.726	0.177
Sibling x Nutrients	4	53.407	13.352	16	15.482	0.862	0.507
Error	16	247.718	15.482				
(B) Nitrogen content							
Nutrients	1	6.676	6.676	3.669	38.545	0.173	0.700
Mesocosm (Nutrients)	4	159.792	39.948	16	5.462	7.314	0.002
Sibling	4	16.647	4.162	4	4.059	1.025	0.491
Sibling x Nutrients	4	16.235	4.059	16	5.462	0.743	0.576
Error	16	87.387	5.462				
(C) CN ratio							
Nutrients	1	< 0.001	< 0.001	4.488	333.810	< 0.001	1
Mesocosm (Nutrients)	4	1226.652	306.663	16	42.265	7.256	0.002
Sibling	4	297.260	74.315	4	69.412	1.071	0.474
Sibling x Nutrients	4	277.649	69.412	16	42.265	1.642	0.212
Error	16	676.237	42.265				

### Upwelling experiment (Mid-September 2014)

#### O_2_, salinity and temperature conditions

The mean (± SD) of O_2_ concentration during the experimental upwelling in the PVC boxes was 2.71 ± 0.37 mg L^-1^ (range 1.44 mg L^-1^ to 5.61 mg L^-1^). Mean temperature during the hypoxia experiment was 16.35 ± 0.29°C (range 15.8°C to 17.5°C). pH was 7.40 and salinity was 22.8 psu in the deep water measured once on the day before the start of the hypoxia treatment.

#### Survival

**Fig 5:**
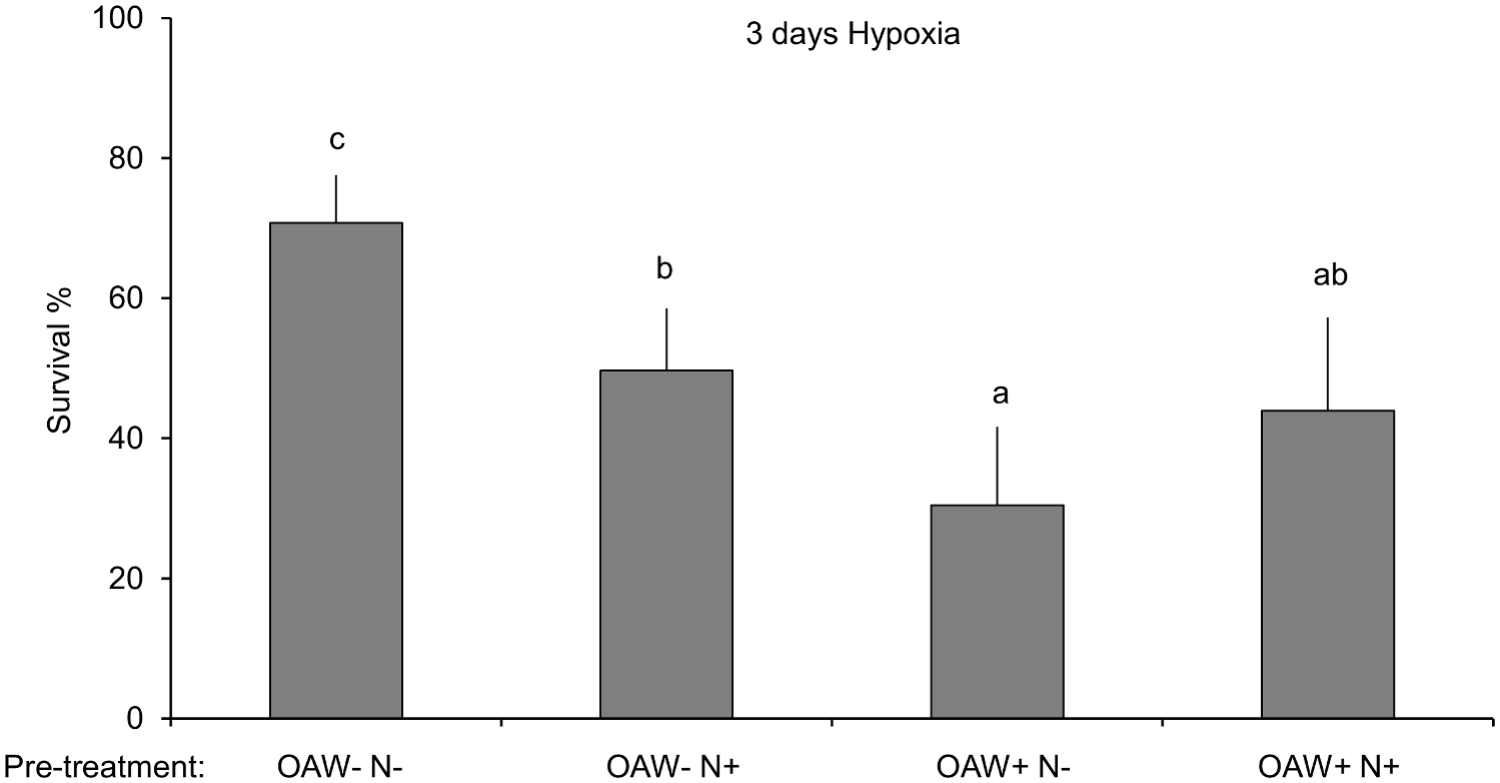
Survival of *F*. *vesiculosus* germlings after 3 days of hypoxic upwelling. Survival (%, mean +SD, n = 3) in 16 sibling groups of *F*. *vesiculosus* germlings previously treated under the four treatment combinations of the OAW x nutrient experiment: OAW-N-, OAW-N+, OAW+ N-, OAW+N+. Different letters above the bars indicate significant differences (p-value < 0.05) between the treatments after Tukey’s HSD.

In all sibling groups with different treatment histories, the three days of hypoxia reduced germling survival significantly. Survival was highest for germlings pre-treated at ambient conditions (OAW-N-) and lowest for germlings previously treated at high OAW and ambient nutrients (OAW+N-) (Fig 5). Thus, high OAW significantly doubled the sensitivity to subsequent hypoxia as compared to a non-warmed, non-acidified regime (Split-plot ANOVA, OAW, p-value < 0.05, Fig 5, Table 2C). Nutrient addition during the preceding experiment enhanced sensitivity to hypoxia in germlings stemming from a regime of ambient temperature and CO_2_ conditions but not for those from a OAW+ regime as reflected in the significant interaction between ‘OAW’ and ‘Nutrients’ (Split-plot ANOVA, OAW x Nutrients, p-value < 0.05, Fig 5, Table 2C).

## Discussion

The simulated OAW as expected for 2110 strongly reduced survival and growth of *F*. *vesiculosus* germlings. The analysis of the single factors warming and acidification in a previous study at the KOB showed that warming was the main driver of mortality of *F*. *vesiculosus* germlings, while acidification played a minor role [33]. At temperatures > 27°C, reduction in growth and photosynthetic efficiency were observed in adult *F*. *vesiculosus*, finally resulting in necrosis [34]. Reduced growth of germlings under heat stress (> 25°C) was also observed in this work. This shows that the upper limits of thermotolerance of *F*. *vesiculosus* performance are similar in early and adult life-stages.

However, in our multi-factorial design, the negative effects of warming on germling survival and growth rates were strongly mitigated by high nutrient concentrations. Earlier studies on Baltic adult *F*. *vesiculosus* have shown that nutrient enrichment increases nutrient uptake [65] and enhances photosynthetic efficiency [27]. Similar responses were also observed in other algal species. *Ulva rigida* cultured under nutrient enrichment reacted with higher nitrogen uptake, higher nitrate reductase activity and higher growth rates. Moreover, the nitrogen reductase activity was enhanced under future (1000 μatm) compared to ambient pCO_2_ (400 μatm) conditions [66]. Similarly, high pCO_2_ enhanced nitrogen assimilation in the brown alga *Hizikia fusiforme* [67] and may have decreased the relative investment in the nitrogen-intensive protein biosynthesis [67]. Consequently, nitrogen may have been freed for other processes, such as growth and nitrogen storage [68]. Possibly, the *F*. *vesiculosus* germlings in our experiments also took up more nitrogen under nutrient enrichment when additional CO_2_ was provided under acidified (and warmed) conditions, resulting in increased growth and survival. Our experimental design did not allow for disentangling the different possible mechanisms of mitigating effects of nutrients on either warming or acidification. Photosynthesis is regarded as one of the most heat sensitive metabolic activities in the plant cell [69, 70], with at least three major heat-stress sensitive sites in the photosynthetic machinery: the photosystems (mainly photosystem II with its oxygen-evolving complex), the ATP generating and the carbon assimilation processes [71]. Moreover, respiration rates are increased under warming [72]. As our response variables growth and survival represent responses integrating over many metabolic processes, several compensatory effects caused by high nutrient levels appear possible. In conclusion, nutrient enrichment compensated to some degree the severe negative effects of future heat stress on *F*. *vesiculosus* germlings, which may be further mitigated by higher carbon availability under acidified conditions. However, such direct beneficial effects of nutrient enrichment may be overridden by indirect detrimental effects of eutrophication (such as increases in water turbidity, sedimentation, grazing and abundance of epibiotic filamentous algae) at field conditions, as reviewed by Berger et al. [45]. During the OAW x nutrient experiment, the epibiota under ambient and enriched nutrient conditions have not been determined. Regular filtration of the water content of our experimental boxes kept the fouling load relatively low under both, ambient and high nutrient conditions, assuming that epibiota had no strong effects on the *F*. *vesiculosus* germlings.

Increased nitrogen uptake is accompanied by higher CO_2_ uptake (even at ambient pCO_2_), hence a constant CN ratio is maintained [66]. Our findings show that the CN ratio in *F*. *vesiculosus* germlings was lower in the high nutrient treatment. Although this difference was not significant, it suggests higher uptake rates of nitrogen under nutrient enrichment. Since the nitrogen concentration in Baltic *F*. *vesiculosus* thalli is lowest in summer [73], nutrient enrichment effects may be most conspicuous in this season. This may have contributed to the observed stress-mitigating effect of nutrient enrichment in *F*. *vesiculosus* germlings. The buffering of stress impact by additional resources was also observed in juvenile blue mussel *Mytilus edulis*, when high food conditions enhanced the tolerance to ocean acidification [74].

The different sibling groups showed high variations in survival under warming and acidification, indicating the enhanced potential for adaptation in genetically diverse populations [48]. The crucial role in genetic variation for recovery from disturbances has also been reported in estuarine macrophytes [75] allowing for adaptation under global change stress [76].

The three day hypoxia experiment in the KOB simulating an upwelling event induced substantial germling mortality. During a local upwelling event, deep water with low oxygen concentration, low temperatures an increased pCO_2_ and high salinity is shoaling [77, 78]. In our upwelling treatment, temperature did not decrease considerably (16.41 ± 0.33°C) compared to previous values of 19.26 ± 2.38°C. Likewise, salinity (22.8 psu) did increase only slightly relative to the previous condition (Fig 1C) and the elevated CO_2_ (as associated with hypoxic upwelling) has minor effects on germling survival [33] (Fig 1C). This leaves the low oxygen concentration (2.75 ± 0.41 mg L^-1^) during the upwelling event as the most likely driver of germling mortality. Mortality during the three days of upwelling was considerably higher than during the two months of the preceding experiment, illustrating the high susceptibility of *F*. *vesiculosus* germlings to hypoxia. This susceptibility is probably due to reduced respiration rates under dark conditions, as it was also observed under hypoxic conditions in *Cladophora vagabunda* and *Gracilaria tikvahiae* [79]. Reduced respiration rates are accompanied by decreased provision of ATP and biosynthetic precursors leading to higher stress sensitivity [80] and to a reduced metabolism [79]. Susceptibility to hypoxia impacts was highest on germlings which previously experienced warmed and acidified conditions. Thus, the impact of hypoxic upwelling events in the future may be amplified by synchronous OAW. The assumed increased respiration under warming [72] may have further increased the O_2_ debt, which could not be balanced under hypoxic conditions. Consequently, *F*. *vesiculosus* germlings grown under high compared to ambient temperatures were less tolerant to hypoxia. Thus, germlings grown under high nutrient levels experienced higher mortality under hypoxia compared to those grown at low nutrient levels. Zou et al. [68] demonstrated that under high-nitrogen conditions, respiration was enhanced by high CO_2_ compared to ambient CO_2_ conditions in the macroalga *H*. *fusiforme*. Consequently, in algae growing under nutrient enrichment increased respiration might be necessary to support higher maintenance demands (e.g. due to increased RUBISCO contents) and greater uptake of extra nitrogen [68]. This nutrient-driven higher metabolism may have rendered these germlings more susceptible to hypoxia.

In summary, the responses to hypoxia depended on the preceding OAW x nutrient treatments we applied. This suggests that there are different protective mechanisms in *F*. *vesiculosus* germlings that vary with the type of stressor. Future expansions of hypoxic areas in the Baltic Sea [7] will have severe effects on *F*. *vesiculosus* recruitments, as observed in this experiment, as well as on the benthic community in general [81]. We demonstrated that the net impact of global change including warming, acidification, eutrophication and hypoxia may depend on the interaction among these global and regional factors. This finding underscores the importance for analysing the combined effects of multiple stressors and their interconnectivity for accurate predictions of future scenarios [3]. Moreover, the indirect effects of global change may be more significant than the direct effects [13]. Scaling up multiple stressors is crucial for predicting the fate of *F*. *vesiculosus* populations [82].

## Supporting Information

S1 Fig Nutrient concentrations in the experimental boxes. Nutrient concentrations within the experimental germling boxes before (A, B) and after (C, D) the bi-weekly addition of the nutrients (PO_4_, NO_3_, NO_2_) as well as initial NH_4_ conditions in μmol L^-1^ in July and August. Initial nutrient concentrations were measured six times per month in the main KOB tank before the water addition to the boxes. Nutrient concentrations after additions were determined by adding the sum of the initial and additional nutrient concentration.
(TIFF)
