# Supplementary figures and images for: Buffering and Amplifying Interactions among OAW (Ocean Acidification & Warming) and Nutrient Enrichment on Early Life-Stage Fucus vesiculosus L. (Phaeophyceae) and Their Carry Over Effects to Hypoxia Impact
Source: PLoS One. 2016 Apr 4;11(4):e0152948. doi: 10.1371/journal.pone.0152948 (PMC4820272; doi:10.1371/journal.pone.0152948)

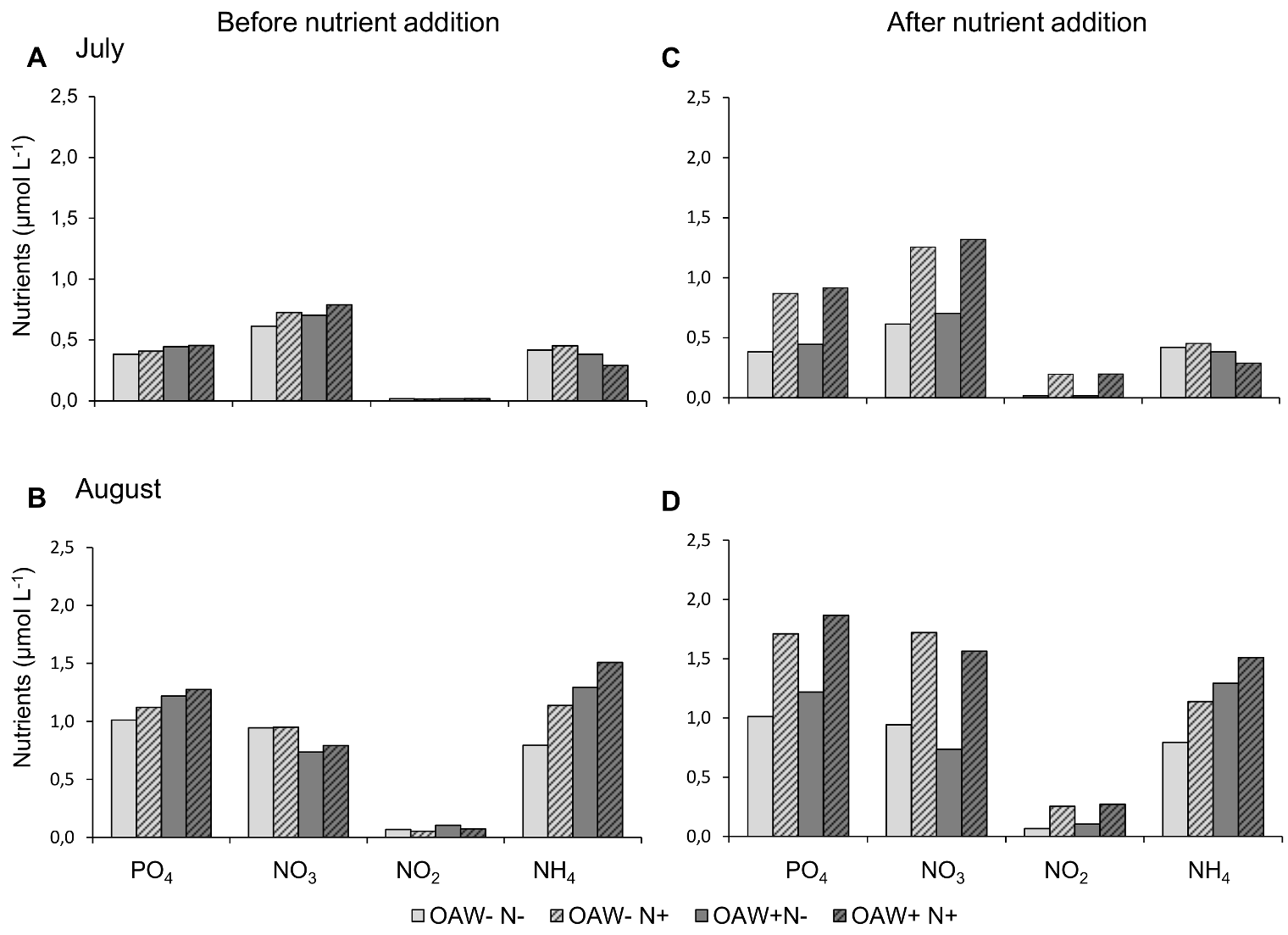

Supplement: S1 Fig — Nutrient concentrations within the experimental germling boxes before (A, B) and after (C, D) the bi-weekly addition of the nutrients (PO4, NO3, NO2) as well as initial NH4 conditions in μmol L-1 in July and August. Initial nutrient concentrations were measured six times per month in the main KOB tank before the water addition to the boxes. Nutrient concentrations after additions were determined by adding the sum of the initial and additional nutrient concentration. (TIFF) [file pone.0152948.s001.tiff]
